# NGS-based targeted sequencing identified two novel variants in Southwestern Chinese families with oculocutaneous albinism

**DOI:** 10.1186/s12864-022-08597-3

**Published:** 2022-04-29

**Authors:** Yuanyuan Xiao, Cong Zhou, Hanbing Xie, Shuang Huang, Jing Wang, Shanling Liu

**Affiliations:** 1 Department of Obstetrics and Gynecology, West China Second University Hospital of Sichuan University, Chengdu, 610041 China; 2 Key Laboratory of Birth Defects and Related Diseases of Women and Children, Ministry of Education, Sichuan University, Chengdu, 610041 China

**Keywords:** Oculocutaneous albinism, *TYR*, *OCA2*, Targeted NGS, Medical genomics, Nucleotide variations

## Abstract

**Background:**

Oculocutaneous albinism (OCA) is a group of heterogeneous genetic diseases characterized by a reduction or complete lack of pigmentation in the hair, skin, and eyes. It is associated with reduced visual acuity, nystagmus, photophobia, and strabismus. OCA type 1 (OCA1) and type 2 (OCA2) are caused by mutations in the tyrosinase (*TYR*) and *OCA2* genes, which are responsible for most cases of OCA. The present study aimed to identify the mutational spectra of 18 southwest Chinese probands with OCA.

**Results:**

We used a skin disease-targeted panel to sequence more than 400 genes, including 23 genes (*TYR*, *OCA2*, *AP3B1*, *BLOC1S3*, *BLOC1S6*, *C10orf11*, *DTNBP1*, *FRMD7*, *GPR143*, *HPS1*, *HPS3*, *HPS4*, *HPS5*, *HPS6*, *LYST*, *MC1R*, *MITF*, *MLPH*, *MYO5A*, *RAB27A*, *SLC24A5*, *SLC45A2*, *TYRP1*) associated with syndromic and non-syndromic albinism. The targeted panel was applied to 18 patients from southwest China, nine (50%) patients were diagnosed with OCA1, and nine (50%) were diagnosed with OCA2. Our data indicate that OCA1 and OCA2, the most common subtypes, probably have the same prevalence in southwest China. In total, we identified 26 variants in *TYR* and *OCA2* from 18 OCA cases using the NGS technology, including 24 variants presented in the Human Gene Mutation Database Professional (HGMD) and two novel variants, c.559_560insCATTATTATGTGTCAAATTATCCCC in *TYR* and c.1514 T > C in *OCA2*, which have not been previously reported. According to the American College of Medical Genetics and Genomics (ACMG) classification, c.559_560insCATTATTATGTGTCAAATTATCCCC (p.G190Cfs*12) is classified as a pathogenic variant, and c.1514 T > C (p.F505S) is evaluated as a likely pathogenic variant.

**Conclusions:**

Two novel variants were identified which will expand the mutational spectra of *TYR* and *OCA2*. The results of the present study may have implications for genetic counseling, carrier screening, and clinical management of the disease.

## Background

Oculocutaneous albinism (OCA) is a group of autosomal recessive inherited disorders characterized by a lack of melanin synthesis [1]. Clinical manifestations include partial or complete loss of hypopigmented hair, skin, and eyes and are always accompanied by ocular abnormalities, such as variably reduced visual acuity, nystagmus, photophobia, and strabismus. According to the presence of other symptoms such as bleeding diathesis, immunodeficiency, or neurological dysfunction, OCA is clinically subdivided into syndromic and non-syndromic OCA. Mutations in Hermansky–Pudlak syndrome 1 (*HPS1*), *HPS3*, *HPS4*, *HPS5*, *HPS6*, adaptor related protein complex 3 subunit beta 1 (*AP3B1*), dystrobrevin binding protein 1 (*DTNBP1*), biogenesis of lysosomal organelles complex 1 subunit 3 (*BLOC1S3*), pallidin (*PLDN*), lysosomal trafficking regulator (*LYST*), myosin-Va (*MYO5A*), melanophilin (*MLPH*), or ras-like protein in the brain 27a (*RAB27A*) can cause syndromic OCA [2, 3]. Mutations in tyrosinase (*TYR*), oculocutaneous albinism type 2 (*OCA2*), melanocortin 1 receptor (*MC1R*), tyrosinase-related protein 1 (*TYRP1*), solute carrier family 45 member 2 (*SLC45A2*), solute carrier family 24 member 5 (*SLC24A5*), and chromosome 10 open reading frame 11 (*C10ORF11*) can cause non-syndromic OCA, which can be broadly classified into several subtypes—OCA type 1, OCA type 2, OCA type 3, OCA type 4, OCA type 6, and OCA type 7 [1, 4]. The estimated prevalence of OCA subtypes varies significantly among different ethnic populations. OCA type 1 (OCA1) and OCA type 2 (OCA2) are the most common forms, accounting for approximately 80% of all OCA cases worldwide [1, 5]. In recent years, comprehensive genetic analyses have revealed the subtype distribution of Chinese patients with albinism. Although the proportions of each type are different among the populations from different provinces, OCA1 and OCA2 are considered the most prevalent types [6, 7].

Clinical diagnosis of the OCA subtype is difficult because of the overlapping and variable clinical phenotype. Emerging evidence indicates that next-generation sequencing (NGS), which has become a standard and efficient technology for mutational screening of genes associated with OCA in patients with clinically suspected OCA, is necessary for the accurate identification of causative genes/new subtypes of OCA [8–10]. In the current study, we sequenced more than 400 genes known to cause skin diseases, including 23 genes (*TYR*, *OCA2*, *AP3B1*, *BLOC1S3*, *BLOC1S6*, *C10orf11*, *DTNBP1*, *FRMD7*, *GPR143*, *HPS1*, *HPS3*, *HPS4*, *HPS5*, *HPS6*, *LYST*, *MC1R*, *MITF*, *MLPH*, *MYO5A*, *RAB27A*, *SLC24A5*, *SLC45A2*, *TYRP1*) associated with syndromic and non-syndromic albinism using targeted NGS in 18 patients, and identified 26 mutations in *TYR* and *OCA2*. After database retrieval and literature searches, two novel variants were identified.

## Results

In the present study, we used a skin disease-targeted panel to sequence more than 400 genes, including 23 genes (*TYR*, *OCA2*, *AP3B1*, *BLOC1S3*, *BLOC1S6*, *C10orf11*, *DTNBP1*, *FRMD7*, *GPR143*, *HPS1*, *HPS3*, *HPS4*, *HPS5*, *HPS6*, *LYST*, *MC1R*, *MITF*, *MLPH*, *MYO5A*, *RAB27A*, *SLC24A5*, *SLC45A2*, *TYRP1*) associated with syndromic and non-syndromic albinism. Most of the patients presented with typical OCA manifestations on their skin, hair, and iris. At the same time, the patients did not show any other phenotypes involving other organ systems. The clinical features of the 18 patients are presented in Table 1. Twenty-six variants of *TYR* and *OCA2* were identified in 18 patients, including 24 previously reported and two novel variants (Table 2). Nine patients were diagnosed with OCA type 1 (OCA1) due to *TYR* mutations, and nine patients were diagnosed with OCA type 2 (OCA2) due to *OCA2* mutations.

**Table 1:** Clinical features of the Probands

Patient no	Sex	Age	Skin color	Hair color	Iris color	Amblyopia	Nystagmus	Photophobia
1	M	64 years	White	White	Brown	Positive	Positive	Positive
2	M	5 years	Creamy white	White	Brown	Positive	Positive	Positive
3	M	4 years	White	White	Pink	Positive	Positive	Positive
4	F	1 years	White	White	Little Pink	Positive	Positive	Positive
5	F	8 months	Creamy white	White	Little Pink	Positive	Positive	Positive
6	F	3 years	Creamy white	Light Blond	Little Pink	Positive	Positive	Positive
7	F	5 years	White	White	Little Pink	Positive	Positive	Positive
8	M	6 years	White	White	Blue-gray	Positive	Positive	Positive
9	M	3 years	Creamy white	White	Little Pink	Positive	Positive	Positive
10	M	2 years	Creamy white	Light Blond	Light brown	Positive	Positive	Positive
11	M	8 years	Creamy white	Light Blond	Blue-gray	Positive	Positive	Positive
12	F	28 years	Creamy white	Light Blond	Blue-gray	Positive	Positive	Positive
13	F	2 months	White	White	Blue-gray	Unknown	Unknown	Unknown
14	F	27 years	Lighter than normal	Light Blond	Light brown	Positive	Positive	Positive
15	M	5 years	Creamy white	Light Blond	Blue-gray	Positive	Positive	Positive
16	F	11 months	Creamy white	Light Blond	Blue-gray	Positive	Positive	Unknown
17	M	2 months	Creamy white	Light Blond	Blue-gray	Unknown	Unknown	Unknown
18	F	32 years	Lighter than normal	Blond	Brown	Positive	Positive	Positive

**Table 2:** Variants in 18 probands with Oculocutaneous Albinism

Patient no	Molecular subtype	Gene	Location	Mutation type	Nucleotide Change	Amino Acid Change	Hom/Het
1	OCA1	*TYR*	exon1	nonsynonymous	c.425A > T	p.K142M	het
		*TYR*	exon4	nonsynonymous	c.1193A > G	p.E398G	het
2	OCA1	*TYR*	exon2	stopgain	c.832C > T	p.R278*	hom
3	OCA1	*TYR*	exon1	stopgain	c.346C > T	p.R116*	het
		*TYR*	exon2	nonsynonymous	c.880G > A	p.E294K	het
4	OCA1	*TYR*	exon2	frameshift	c.929dupC	p.R311Kfs*7	het
		*TYR*	exon1	nonsynonymous	c.299G > T	p.C100F	het
5	OCA1	*TYR*	exon4	nonsynonymous	c.1346A > G	p.Y449C	het
		*TYR*	exon1	frameshift	**c.559_560insCATTATTATGTGTCAAATTATCCCC**	p.G190Cfs*12	**het**
6	OCA1	*TYR*	exon1	stopgain	c.346C > T	p.R116*	het
		*TYR*	exon4	nonsynonymous	c.1199G > T	p.W400L	het
7	OCA1	*TYR*	exon2	frameshift	c.929dupC	p.R311Kfs*7	het
		*TYR*	exon1	nonsynonymous	c.230G > A	p.R77Q	het
8	OCA1	*TYR*	exon1	nonsynonymous	c.164G > A	p.C55Y	het
		*TYR*	exon1	nonsynonymous	c.715C > T	p.R239W	het
9	OCA1	*TYR*	exon2	splicing	c.820-3C > G	splicing	het
		*TYR*	exon2	frameshift	c.929dupC	p.R311Kfs*7	het
10	OCA2	*OCA2*	exon23	nonsynonymous	c.2363C > T	p.S788L	het
		*OCA2*	exon23	splicing	c.2339-2A > C	splicing	het
11	OCA2	*OCA2*	exon13	nonsynonymous	c.1349C > T	p.T450M	het
		*OCA2*	exon20	splicing	c.2080-2A > G	splicing	het
12	OCA2	*OCA2*	exon4	stopgain	c.493C > T	p.R165*	hom
13	OCA2	*OCA2*	exon13	nonsynonymous	c.1349C > T	p.T450M	het
		*OCA2*	exon15	nonsynonymous	**c.1514 T > C**	**p.F505S**	**het**
14	OCA2	*OCA2*	exon9	frameshift	c.980dupT	p.E328Rfs*21	het
		*OCA2*	exon13	nonsynonymous	c.1255C > T	p.R419W	het
15	OCA2	*OCA2*	exon11	splicing	c.1182 + 1G > A	splicing	het
		*OCA2*	exon21	nonsynonymous	c.2228C > T	p.P743L	het
16	OCA2	*OCA2*	exon6	nonsynonymous	c.583A > G	p.S195G	het
		*OCA2*	exon13	nonsynonymous	c.1255C > T	p.R419W	het
17	OCA2	*OCA2*	exon14	nonsynonymous	c.1426A > G	p.N476D	het
		*OCA2*	exon4	stopgain	c.493C > T	p.R165*	het
18	OCA2	*OCA2*	exon23	splicing	c.2339-2A > C	splicing	het
		*OCA2*	exon13	nonsynonymous	c.1255C > T	p.R419W	het

Novel mutations are in bold. *Het* Heterozygous, *Hom* Homozygous. RefSeq of the genes used: *TYR*: NM_000372; *OCA2*: NM_000275

### Variants of *TYR*

**Fig. 1:**
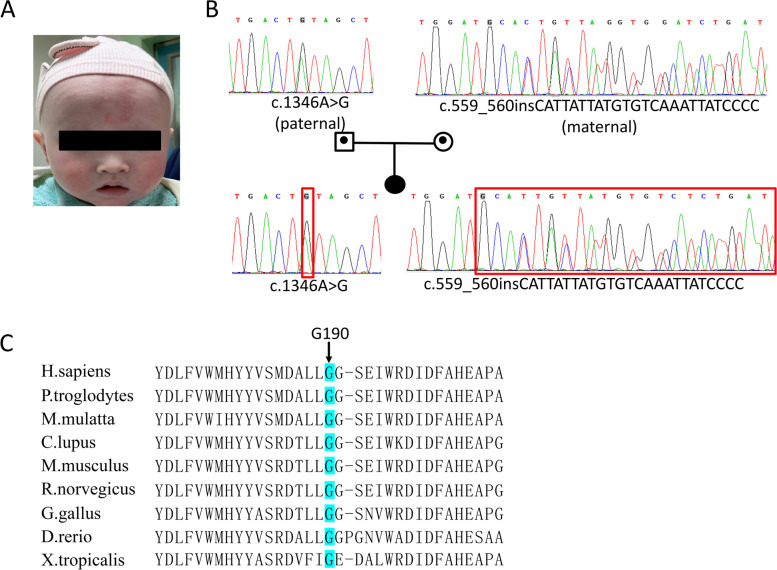
**A** A picture of an OCA patient (Patient.5 in Table 1) with creamy white skin color and white hair color. **B** A pedigree drawing of Sanger sequencing of the OCA patient. The girl inherited the c.1346A > G and c.559_560insCATTATTATGTGTCAAATTATCCCC from father and mother, respectively. **C** The p.G190 in TYR is highly conserved amino acids throughout evolution

Genetic investigation revealed 14 variants, including one novel mutation, among the nine patients affected by *TYR* mutations. Eight patients were compound heterozygous, and one patient was homozygous (Table 2). All patients were from non-consanguineous families. The most common mutation was c.929dupC (p.R311Lfs*7), which accounted for 17.6% of the *TYR* mutations, followed by c.346C > T (p.R116*), accounting for 11.8%. All the other identified mutations occurred in only one patient (Table 2). Patient 5 was compound heterozygous for a previously reported missense (*TYR* c.1346A > G, p.Y449C) mutation and one novel frameshift mutation (*TYR* c.559_560insCATTATTATGTGTCAAATTATCCCC, p.G190Cfs*12) mutation (Fig. 1A-B). This novel variant was absent in the gnomAD, 1000 Genomes Database, the Exome Aggregation Consortium (ExAC), or the Human Gene Mutation Database Professional (HGMD, version 2021.3) and ClinVar databases. p.G190 in *TYR* is a highly conserved amino acid throughout evolution (Fig. 1C). The novel frameshift mutation is additions of 25 nucleotides at c.559_560 of exon 1, which leads to a frameshift at codon 190 with a premature chain termination codon at position 201. According to the American College of Medical Genetics and Genomics (ACMG) classification, c.559_560insCATTATTATGTGTCAAATTATCCCC (p.G190Cfs*12) is classified as a pathogenic variant with one very strong (PVS1), one moderate (PM2), and one supporting (PP4) pathogenicity evidence (Table 3).

**Table 3:** Bioinformatic analysis of the two novel mutations

Gene	Nucleotide Change	Amino acid change	SIFT_Predict	PolyPhen_2_Predict	MutationTaster_Predict	Conservation	ACMG classification
*TYR*	c.559_560insCATTATTATGTGTCAAATTATCCCC	p.G190Cfs*12	NA	NA	NA	Conserved	PVS1,PM2,PP4 pathogenic
*OCA2*	c.1514 T > C	p.F505S	Damaging	Probably_damaging	Disease_causing	Conserved	PM2,PM3,PP3,PP4 likely pathogenic

SIFT_Predict classification: damaging, tolerated; PolyPhen_2_Predict classification: damaging, probably damaging, benign; MutationTaster_Predict classification: disease-causing, polymorphism. ACMG classification: pathogenic, likely pathogenic, uncertain significance, likely benign and benign

*NA* Not available

### Variants of *OCA2*

**Fig. 2:**
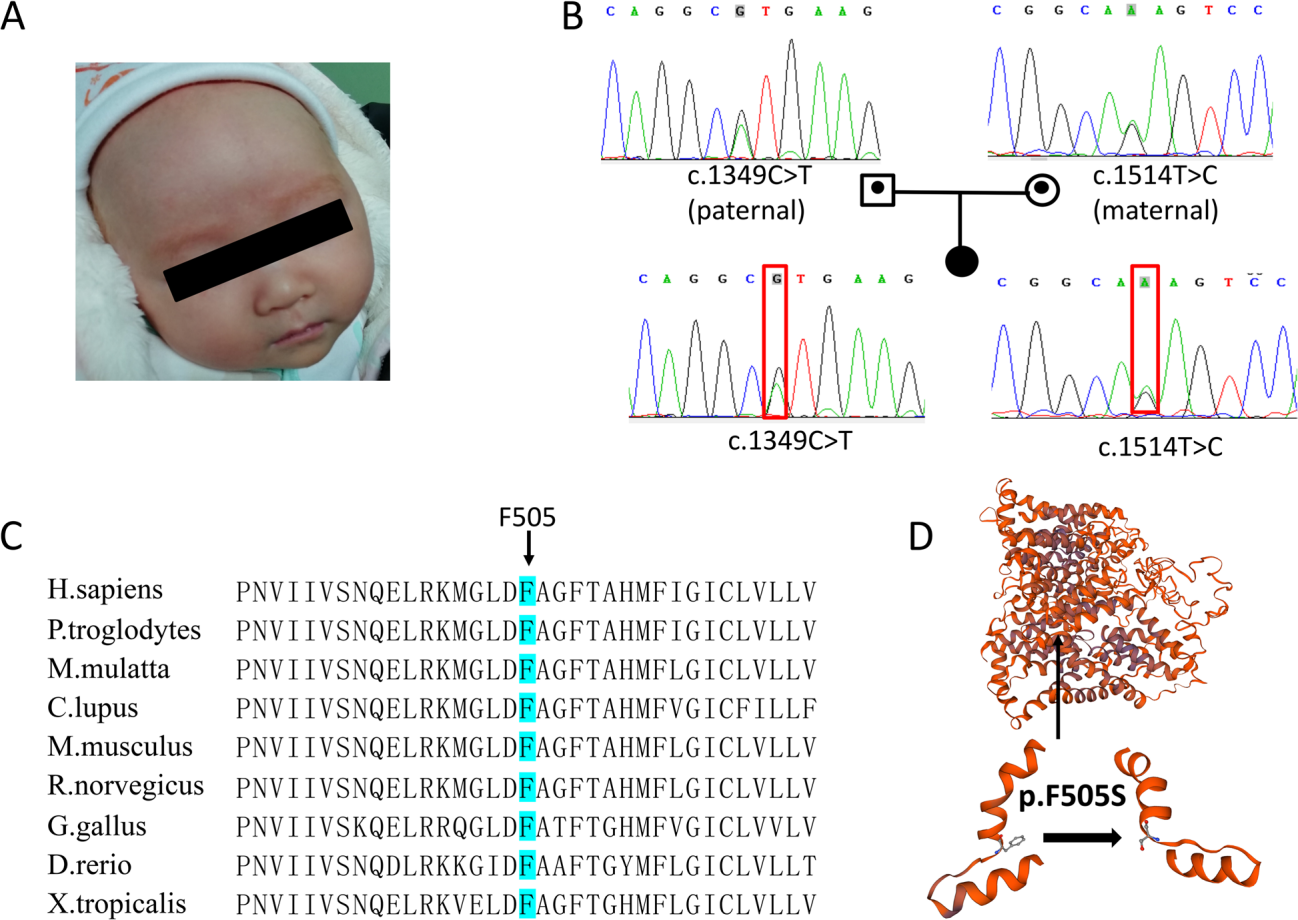
**A** A picture of an OCA patient (Patient.13 in Table 1) with white skin color and white hair color. **B** A pedigree drawing of Sanger sequencing of the OCA patient. The girl inherited the c.1349C > T and c.1514 T > C from father and mother, respectively. **C** The p.F505 in OCA2 is highly conserved amino acids throughout evolution. **D** Modeling of the amino acids conformation changes by SWISS-MODEL

Nine OCA2 patients were genotyped, eight of which were compound heterozygous and one possessed homozygous variants, as shown in Table 2. The patients with homozygous variants were from the consanguineous family, and her brother presented with similar cutaneous and ophthalmic features. Twelve different *OCA2* variants, including one novel mutation, were identified. The most common mutation was c.1255C > T (p.R419W), which accounted for 17.6% of the *OCA2* mutations, followed by c.1349C > T (p.T450M), c.2339-2A > C (splicing), and c.493C > T (p.R165*), accounting for 11.8%. All other identified mutations were detected in only one patient (Table 2). Patient 13 was compound heterozygous for a previously reported missense (OCA2 c.1349C > T, p.T450M) mutation and one novel missense mutation (OCA2 c.1514 T > C, p.F505S) (Fig. 2A,B). This novel variant was not reported in the HGMD Professional (version 2021.3), ClinVar database and 1000 Genomes Database, and it was as rare in the new version and only in couple of databases (gnomAD_exome_all and ExAC_all). This rare novel variant in the OCA2 protein is located at an amino acid position that was highly conserved throughout evolution (Fig. 2C). Phenylalanine (F) has Cs and Hs in its aromatic side chain, it is hydrophobic and nonpolar, and prefers to be buried in protein hydrophobic cores. Serine (S) is polar and has hydrophilic side chain. The amino acid substitution has important implications for proteins' tertiary structure and may cause potentially deleterious effects. Significant structural changes in amino acid composition and polypeptides caused by the variants were observed through the simulation of SWISS‐MODEL (Fig. 2D). According to ACMG classifications, c.1514 T > C (p.F505S) was evaluated as a likely pathogenic variant with two moderate (PM2 and PM3) and two supporting (PP3 and PP4) pathogenicity evidence (Table 3).

## Discussion

As the clinical manifestations of different OCA subtypes are similar, it is difficult to make a differential clinical diagnosis for OCA subtyping. Molecular and genetic analyses are useful for disease classification and genetic counseling. Sanger sequencing, as the clinical gold standard technique, was used to identify candidate variants of albinism genes in most clinical genetic laboratories. With the development of technology, NGS has become an efficient, accurate, high-throughput, and relatively low-cost method that can simultaneously screen all known genes related to OCA.

To date, many variants associated with OCA have been identified, and the mutational spectra of these genes differ among racial and ethnic populations [11]. In America, *OCA1* and *OCA2* are the most common forms, with prevalence rates of 69% and 18%, respectively [12]. In Italy, the prevalence of *OCA1* and *OCA2* is 73.3% and 13.3%, respectively [11]. In India, the prevalence rates of *OCA1* and *OCA2* are 59.8% and 10.9%, respectively [13]. *OCA1* (69%) and *OCA4* (23%) are the most frequent forms in Hungary [14]. *OCA1* is the most common variant in Caucasians [12]. However, in Africa, the most frequent variant is *OCA2* [15].

In the present study, nine (50%) patients were diagnosed with OCA1, and nine (50%) were diagnosed with OCA2. This was different from a previous study (70.1% and 10.2%, respectively) in Chinese people, although the previous study involved 127 OCA patients who were from more than 20 different provinces [6]. Our data indicate that OCA1 and OCA2, the most common subtypes, probably have the same prevalence in southwest China. Additionally, we identified 26 variants in *TYR* and *OCA2* from 18 OCA cases using the NGS technology (Table 2), in addition to two novel variants, c.559_560insCATTATTATGTGTCAAATTATCCCC in *TYR* and c.1514 T > C in *OCA2*, which have not been previously reported.

A total of 14 different *TYR* mutations were identified in this study: nine missense mutations (c.425A > T, c.1193A > G, c.880G > A, c.299G > T, c.1346A > G, c.1199G > T, c.230G > A, c.164G > A, and c.715C > T), two nonsense mutations (c.832C > T, c.346C > T), two insertion mutations (c.929dupC, c.559_560insCATTATTATGTGTCAAATTATCCCC), and one splicing mutation (c.820-3C > G). The insertion mutation, c.929dupC, located on *TYR* exon2, was the most frequent *TYR* mutation in our study, which is consistent with the data from Chinese and other East Asian countries, such as Korea and Japan [16–20]. The insertion mutation c.559_560insCATTATTATGTGTCAAATTATCCCC, located on *TYR* exon1, has not been previously reported, following the American College of Medical Genetics and Genomics (ACMG) standards and guidelines for the interpretation and reporting of sequence variants, this variant was classified as “pathogenic” (Table 3). Missense mutations (64.3%, 9/14) in this study were the most frequent type in OCA1 patients, which is consistent with previous reports [20, 21].

Meanwhile, a total of 12 different OCA2 mutations were identified in the present study, including seven missense mutations (c.2363C > T, c.1349C > T, c.1514 T > C, c.1255C > T, c.2228C > T, c.583A > G, c.1426A > G), three splicing mutations (c.2339-2A > C, c.2080-2A > G, c.1182 + 1G > A), one frameshift mutation (c.980dupT), and one nonsense mutation (c.493C > T). The missense mutation c.1514 T > C has not been previously reported. Using the in-silico prediction tools (Sorting Intolerant From Tolerant (SIFT), Polymorphism Phenotyping v2 (Polyphen-2), and Mutation Taster), it was determined that c.1514 T > C exerts possible deleterious effects on protein structure, stability, and function. Following the ACMG standards and guidelines for the interpretation of sequence variants, c.1514 T > C was classified as a “likely pathogenic” variant (Table 3).

## Conclusions

In conclusion, we identified 26 variants, including two novel variants, in OCA patients from southwest China using the NGS technology. Our results enlarged the mutational spectra of *TYR* and *OCA2*, and the identification of novel pathogenic or likely pathogenic variants of *OCA* will benefit gene diagnostics and genetic counseling for OCA patients. Moreover, our study indicates that the targeted NGS technology is an effective molecular diagnostic method for mutational screening of patients with clinically suspected OCA.

## Methods

### Patients and clinical data

From July 2017 to November 2020, we recruited 18 OCA patients between 2 months and 63 years of age, at the West China Second University Hospital of Sichuan University (Chengdu, China). The 18 patients visited the department of medical genetics of West China Second University Hospital for genetic consultation. The typical clinical characteristics of OCA, including poor eyesight, photophobia and nystagmus in the eyes, and white or little blond hair or skin, were observed in most patients. Seventeen patients were from non-consanguineous families, and one patient was from a consanguineous family in Sichuan Province. This study was approved by the Medical Ethics Committee of West China Second University Hospital of Sichuan University for experiments involving humans. Written informed consent was obtained from all participants or their legal guardians. Clinical information for the 18 patients with OCA in this study is shown in Table 1.

### Genetic analysis

Peripheral blood samples were collected from the patients and their parents in tubes containing ethylenediaminetetraacetic acid (EDTA), and genomic DNA was isolated using the QIAamp DNA Blood Mini Kit (QIAGEN, Hilden, Germany) according to the manufacturer's protocol [22]. DNA concentration and purity were determined using a NanoDrop 2000 UV–vis spectrophotometer (Termo Fisher Scientific, Massachusetts, United States). Patient genotypes were established through NGS with a panel of genes involved in OCA using the Targeted Exome Capture Kit (MyGenostics, Beijing, China) according to the manufacturer’s instructions [23]. The kit targets more than 400 genes known to cause skin diseases, including 23 genes (*TYR*, *OCA2*, *AP3B1*, *BLOC1S3*, *BLOC1S6*, *C10orf11*, *DTNBP1*, *FRMD7*, *GPR143*, *HPS1*, *HPS3*, *HPS4*, *HPS5*, *HPS6*, *LYST*, *MC1R*, *MITF*, *MLPH*, *MYO5A*, *RAB27A*, *SLC24A5*, *SLC45A2*, *TYRP1*) associated with syndromic and non-syndromic albinism. NGS was performed using the NextSeq 500 platform (Illumina Inc, San Diego, United States) that captured all exons of the targeted genes with intronic 50 bp flanking sites. The sequence reads were aligned to the human reference genome (UCSC GRCh37/hg19) using the Burrows-Wheeler Aligner. The variants were called using Genome Analysis Tool Kit, and were annotated using ANNOVAR. All variants were then further checked by the Genome Aggregation Database (gnomAD, http://gnomad.broadinstitute.org/), the Exome Aggregation Consortium (ExAC, http://exac.broadinstitute.org/), Exome Sequencing Project v. 6500 (Esp6500, http://evs.gs.washington.edu/EVS), the 1000 Genomes Database (http://browser.1000genomes.org/index.html). Variants with Minor allele frequency (MAF) of < 0.05 were retained. Effects of the variants were assessed in silico. Sorting Intolerant From Tolerant (SIFT, http://sift.jcvi.org/), Polymorphism Phenotyping v2 (Polyphen-2, http://genetics.bwh.harvard.edu/pph2/), and Mutation Taster (http://www.mutationtaster.org/) were used to identify the potentially deleterious effects of amino acid substitution on its structure and function. The conservative analysis of single amino acid sites was performed as reported in Homologene (https://www.ncbi.nlm.nih.gov/homologene), and the conformational protein changes caused by amino acid substitutions were simulated via SWISS-MODEL (https://swissmodel.expasy.org). Finally, the pathogenic variants were compared with the Human Gene Mutation Database (HGMD®, http:// portal.biobase-international.com/ghmd/pro/search_gene.php) and ClinVar database (http://www.ncbi.nlm.nih.gov/clinvar) to determine whether they were reported or novel variants. The nomenclature of novel variants was based on the international gene variant nomenclature system proposed by the Human Genome Variant Society (HGVS, http://www.hgvs.org/mutnomen).

### Validation by Sanger sequencing

All suspected variants were confirmed and checked for inheritance of the variant in trans by Sanger sequencing within each patient’s family. Primers were designed using the gene tool software to amplify specific regions containing variants using polymerase chain reaction (PCR). The PCR products were directly sequenced using an ABl3730 Genetic Analyzer (Applied Biosystems, California, United States). Sanger sequencing data were analyzed using the Chromas software (Technelysium, South Brisbane, Australia). Additionally, variants were rigorously cataloged as pathogenic, likely pathogenic, or of unknown clinical significance by following the interpretation guidelines of the American College of Medical Genetics and Genomics (ACMG) [24].

## Data Availability

The raw datasets used and analysed during the current study are not deposited in publicly available repositories because of considerations about the security of human genetic resources. The genetic mode and disease information of relevant genes in this article can be obtained from the OMIM database: https://www.omim.org/. For other details of the availability of data and material, please refer to the methods section of the article and tables. Sequencing dataset can be obtained from the corresponding author on reasonable request.

## Abbreviations

OCA: Oculocutaneous albinism
TYR: Tyrosinase
NGS: Next-generation sequencing
SIFT: Sorting Intolerant From Tolerant
Polyphen-2: Polymorphism Phenotyping v2
ACMG: American College of Medical Genetics and Genomics
OCA1: OCA type 1
OCA2: OCA type 2
EDTA: Ethylenediaminetetraacetic acid
gnomAD: Genome Aggregation Database
ExAC: Exome Aggregation Consortium
Esp6500: Exome Sequencing Project v. 6500
HGMD: Human Gene Mutation Database
HGVS: Human Genome Variant Society
PCR: Polymerase chain reaction
Het: Heterozygous
Hom: Homozygous
